# Microbial coinfection keratitis: clinical spectrum, pathogen distribution, and antimicrobial susceptibility in an 8-year retrospective study

**DOI:** 10.3389/fmicb.2025.1648962

**Published:** 2025-11-27

**Authors:** María F. Acosta-González, Luis Antonio Bautista-Hernández, Diana Gabriela Ponce-Angulo, Fabiola Anaya-Barragán, Victor Manuel Bautista-de Lucio

**Affiliations:** 1 Research Unit, Department of Microbiology and Ocular Proteomics, Institute of Ophthalmology “Conde de Valenciana Foundation”, Mexico City, Mexico; 2 Postgraduate Program in Health Science, Medical School, National Polytechnic Institute, Mexico City, Mexico; 3 Department of Cornea, Institute of Ophthalmology “Conde de Valenciana Foundation”, Mexico City, Mexico

**Keywords:** keratitis, coinfection, bacterial keratitis, fungi keratitis, antimicrobial resistance, *Staphylococcus epidermidis*, *Fusarium*

## Abstract

**Background:**

Coinfection keratitis, involving concurrent infections by bacteria and fungi, poses diagnostic and therapeutic challenges. This study aimed to describe the microbiological and clinical characteristics of keratitis co-infections, including prevalence, predisposing factors, treatment and antimicrobial susceptibility profiles, in patients from a tertiary ophthalmology referral center in Mexico City.

**Methods:**

This retrospective and descriptive study analyzed data collected over an 8-year period (2012–2020) following STROBE guidelines. Corneal samples from patients diagnosed with infectious keratitis were cultured. Only cases with confirmed coinfections-defined as the isolation of more than one microorganism-were included. Microbiological identification and antimicrobial susceptibility were analyzed. Clinical data included demographics, risk factors, visual acuity, corneal reepithelialization, and surgical interventions.

**Results:**

A total of 306 microorganisms were isolated from 140 patients. Among them, 109 cases (78%) involved bacterial-bacterial coinfections, and 31 cases (22%) were bacterial-fungal coinfections. *Staphylococcus epidermidis* was the most frequently isolated bacterial species (28%), and *Fusarium* was the predominant fungal isolate (52%). Resistance to erythromycin (52%), clindamycin (46%), and fluoroquinolones (35%) were observed, particularly among *S. epidermidis* isolates. Previous ocular surgery (34%) and diabetes mellitus (20%) were the most common risk factors. Similar dominant species were observed among institutional monomicrobial isolates analyzed for contextual reference.

**Conclusion:**

This retrospective and descriptive study characterizes microbial coinfections in infectious keratitis and provides contextual data from microbial cases from the same institution. Coinfection often involves multidrug-resistant organisms and occurs in patients with risk factors such as prior ocular surgery, contact lens use, or diabetes. Early identification and targeted therapy remain essential to improve clinical outcomes.

## 1 Introduction

Microbial keratitis is a serious corneal infection that can lead to ulceration, scarring, perforation, and visual loss if not diagnosticated and treated promptly. While bacteria keratitis is more commonly reported in industrialized nations, fungal keratitis predominates in tropical and resource limited regions. Known risk factors for microbial keratitis include contact lens (CL) wear, corneal trauma, ocular surface disease, topical corticosteroid use, and prior corneal surgery. Systemic conditions such as diabetes mellitus and immunodeficiency also increase susceptibility, and recent evidence suggests that patients with autoimmune disease may be more prone to developing polymicrobial infections (Bograd et al., 2019; González-Dibildox et al., 2021). Coinfection involving two or more microbial agents are relatively uncommon but clinically significant. They can arise from simultaneous infection or secondary colonization following an initial insult (Ahn et al., 2011). These polymicrobial infections are often underdiagnosed due to limitations in culture methods or overlapping clinical features. The presence of multiple organisms complicates management due to varying antibiotic susceptibility and treatment responses (Ponce-Angulo et al., 2020). Inappropriate antibiotic use and prolonged therapy have led to increasing resistance among ocular pathogens, particularly in coinfection settings (Thirumalmuthu et al., 2019). Mixed keratitis can also present diagnostic challenges and lead to poor outcomes if not promptly identified and treated (Bograd et al., 2019; Ahn et al., 2011).

Previous studies on polymicrobial keratitis have primarily been limited to case reports or small series, with limited generalizability (Slade et al., 2008; Singh et al., 2020; Nunes et al., 2016; Lee et al., 2010; Mauger et al., 2010; Sharma et al., 2013; Hong et al., 2014; Linton et al., 2018; Pak et al., 2022). However, recent studies have highlighted the relevance of polymicrobial keratitis in various clinical settings. For example, a community hospital in San Francisco (2019–2021) reported that 25% (44 out of 174) of infectious keratitis cases had a polymicrobial etiology, more frequently observed in vulnerable populations and associated with multidrug-resistant organisms, although clinical outcomes were not significantly worse (Chan et al., 2023). Similarly, a retrospective study by Harbiyeli et al. (2021) found a 6.7% prevalence among 649 cases, with bacteria–fungus combinations being the most common. In that study, larger corneal infiltrates and older age were linked to treatment failure and worse prognosis. Likewise, a cohort from India analyzing 65 patients (2016–2019) reported a 1.36% incidence of polymicrobial keratitis (Rathi et al., 2023), predominantly bacterial–fungal, with a high rate of fluoroquinolone resistance (57%). Overall, medical therapy often failed, leading to early surgical intervention in more than half of the cases.

These studies collectively highlight the importance of understanding coinfection interactions, resistance patterns, and clinical outcomes in corneal infections-particularly in settings with high microbial diversity and variable resource availability-reinforcing the rationale for our eight-year retrospective analysis. The purpose of this study was to analyze the microbiological profile, risk factors, treatment, and antibiotic susceptibility patterns of bacterial and fungal co-infections in keratitis over an 8-year period at a tertiary referral ophthalmology center in Mexico City. Our findings aim to support early clinical suspicion of mixed infections and guide more effective empirical and targeted treatments in at-risk patients.

## 2 Materials and methods

### 2.1 Study design and ethics approval

This retrospective observational study was conducted from 2012 to 2020 at the Institute of Ophthalmology ¨Conde de Valenciana Foundation¨ in Mexico City. Microbiological data were obtained from the Department of Microbiology and Ocular Proteomics of the same institution. The study was approved by the institution’s Research Ethics Committee and followed the tenets of the Declaration of Helsinki. The reporting of results adheres to the STROBE guidelines (Cuschieri, 2019).

### 2.2 Inclusion and exclusion criteria

**Figure 1:**
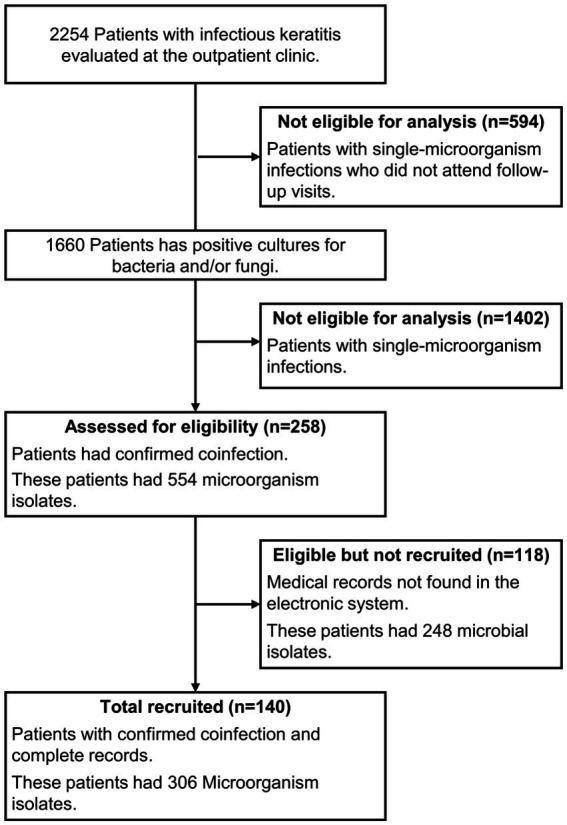
Flowchart of patient selection and inclusion. According to Strengthening the Reporting of Observational studies in Epidemiology guidelines (STROBE) for the choice of patients and the number of microorganisms found. From a total of 2,254 patients with infectious keratitis evaluated at the outpatient clinic. 140 were included after confirmation of microbial coinfection (bacterial and/or fungal).

Patients with clinically suspected infectious keratitis-defined as corneal epithelial defect with stromal inflammation and/or hypopyon-were included if microbiological cultures identified more than one microorganism (i.e., coinfection). Patients with negative cultures or single-organism infections were excluded. Only microorganisms isolated directly from corneal ulcers in patients with compatible clinical findings were considered true pathogens; therefore, potential contaminants or commensals were included only when associated with clinically significant keratitis. The selection process is shown in (Figure 1).

### 2.3 Clinical data collection

Data were extracted from patient records including demographics (age, gender), predisposing ocular and systemic factors (such as trauma, contact lens use, diabetes mellitus, prior herpetic keratitis, and ocular surgery), duration of symptoms at presentation, and clinical features (ulcer location, size, presence of hypopyon, infiltrate, or perforation). Visual acuity was recorded at presentation and at last follow-up, medication use before and after diagnosis, time to corneal re-epithelialization and need for surgical therapy.

### 2.4 Microbiological sampling and culture methods

Corneal samples were taken using sterile cotton swabs from the ulcer margins and base of ulcer after instillation of topical anesthetic streaked sequentially onto chocolate agar, blood agar, Sabouraud dextrose agar, and brain heart infusion (BHI) media. Bacterial cultures were incubated at 37 °C ± 2 °C with 5% CO_2_, while fungal cultures were incubated at 25 °C ± 2 °C. Preliminary results were reviewed after 24 h, with final sensitivity testing performed after 48–72 h. Cultures were considered positive if growth of the same organism was demonstrated on 2 or more solid media; or there was semiconfluent growth at the site of inoculation on 1 solid medium. Gram and Giemsa stains were obtained in every sample, however, a positive microscopy with negative culture was considered as insufficient evidence for microorganism growth. Fungal and slow-growing organisms were incubated for up to 5 days.

### 2.5 Microorganism identification and susceptibility testing

Microbial identification and antibiotic susceptibility were assessed using the semi-automated VITEK 2C® system (bioMérieux, France). Antibiotic groups tested included: fluoroquinolones, aminoglycosides, macrolides, glycopeptides, oxazolidinones, tetracyclines, penicillin’s, cephalosporins, carbapenems, polypeptides and according to Clinical and Laboratory Standards Institute (2007). Medical treatment success was defined as clinical resolution of infection without the need for surgical intervention. Initial treatment failure was defined as ulcer progression, corneal perforation, or need for surgical intervention.

### 2.6 Statistical analysis

Statistical analysis was performed using GraphPad Prism version 5.01 (GraphPad Software Inc. La Jolla, CA). Descriptive statistics included means and standard deviations. The Fisher’s exact test was used to compare categorical variables (e.g., treatment success vs. failure). A paired *t*-test or Mann–Whitney U test, depending on normality compared (e.g., pre- and post-treatment visual acuity (logMAR)). A *p*-value <0.05 was considered statistically significant.

## 3 Results

### 3.1 Patient demographics and clinical characteristics

A total of 140 patients met the inclusion criteria for microbial coinfection in infectious keratitis (Table 1). The cohort included 69 males (49%) and 71 females (51%), with a mean age of 52.1 ± 21.9 years (range: 1–97). The distribution by age was as follows: ≤20 years (10%), 21–40 years (22%), 41–60 years (29%), 61–80 years (30%), and >80 years (9%). The mean time from symptom onset to microbiological diagnosis was 121.1 ± 483.9 days. Prior ocular surgery was documented in 47 patients (34%), particularly among those aged 61–80. Contact lens use was noted in 27 patients (19%), mainly among individuals aged 41–60 years. Systemic comorbidities included diabetes mellitus in 28 patients (20%), with no difference between the groups from 41 and 60 years of age and 61 and 80 years of age, and a history of microbial keratitis in 27 patients (19%), between the ages of 21 and 40.

**Table 1:** Comparison of clinical characteristics and outcomes in patients with bacterial-bacterial vs. bacterial-fungal coinfection.

Variable	Group 1 Bacterial-bacterial (*n* = 109)	Group 2 Fungal-bacterial (*n* = 31)	*p*-value (1 vs. 2)
Sex ^§^			0.542
Males	52 (48%)	17 (55%)	
Females	59 (54%)	14 (45%)	
Age (years) ^¶^			
<20	8 (7%)	3 (10%)	0.411
21–40	26 (24%)	5 (16%)	0.535
41–60	34 (31%)	8 (26%)	0.789
61–80	33 (30%)	10 (32%)	1.000
>80	8 (7%)	3 (10%)	0.141
Mean age	50.8 ± 21.1	52.4 ± 20.9	
Predisposing factors			
Contact lens use ^§^	24 (22%)	3 (10%)	0.195
Diabetes mellitus ^§^	24 (22%)	4 (13%)	0.318
Autoimmune disease ^§^	10 (9%)	1 (3%)	0.455
Previous herpetic keratitis ^§^	8 (7%)	1 (3%)	0.683
Prior microbial keratitis ^§^	15 (14%)	5 (16%)	0.773
Prior ocular surgery ^§^	66 (61%)	11 (35%)	0.015
Ocular trauma			
Chemical burn ^§^	3 (3%)	0	1.000
Non-plant foreign body ^§^	6 (6%)	2 (6%)	1.000
Plant material exposure^§^	5 (5%)	1 (3%)	1.000
Symptom duration (days) ^¶^	53.58 ± 101.95	39.62 ± 69.15	0.086
Poor vision at presentation ^§^	28 (26%)	29 (94%)	**<0.0001**
Initial visual acuity (logMAR) ^¶^	1.41 ± 1.08	1.71 ± 0.87	0.267
Final visual acuity (logMAR) ^¶^	1.53 ± 1.05	2.04 ± 0.82	0.111
Ulcer characteristics			
Central location ^§^	60 (55%)	17 (55%)	1.000
Ulcer size (mm2) ^¶^	3.34 ± 2.18	4.50 ± 3.19	0.194
Size >15 mm^2 §^	3 (3%)	1 (3%)	1.000
Hypopyon ^§^	35 (32%)	5 (16%)	0.114
Surgical treatment			
Penetrating keratoplasty (PKP) ^§^	9 (8%)	3 (10%)	0.728
Other surgery ^§^	11 (10%)	5 (16%)	0.348
Evisceration ^§^	3 (3%)	3 (10%)	0.122
Initial treatment failure ^§^	38 (35%)	15 (48%)	0.209

Comparison between patients with bacterial-bacterial and bacterial-fungal coinfections. § Fisher’s or Chi-square exact test; ^¶^Unpaired *t*-test or Mann–Whitney U test, depending on normality. logMAR: Logarithm of the Minimum Angle of Resolution. Statistically significant (*p* < 0.05). Percentages are calculated per group. Missing data was excluded from analysis when applicable. Bold values indicate statistically significant results (*p* < 0.05).

Lesions were located centrally in 49% of cases, paracentrally in 12%, and peripherally in 14%. Hypopyon was noted in 40 patients (29%), and corneal perforation occurred in 7 patients (5%). Infiltrates were observed in 43 cases (31%). The right eye was affected in 65 cases (46%), and the left eye in 75 cases (54%).

The seasonal distribution of microbial keratitis was as follows: 42 cases in spring (30%), 31 cases in summer (22%), 33 cases in autumn (24%), and 34 cases in winter (24%). Sex- and age-related seasonal trends were identified among the coinfection cases. Female patients predominated in spring (35%), followed by summer (24%), whereas male patients were more frequently affected in autumn (29%) and spring (26%). In terms of age distribution, individuals under 20 years of age occurred mainly in spring (50%); those aged 21–40 years were most affected in autumn (35%); patients aged 41–60 years during summer (34%); the 61–80 age group showed equal distribution in spring and summer (26% each); and patients older than 80 years exhibited a spring predominance (42%).

### 3.2 Microbiological findings

A total of 306 microorganisms were isolated from the 140 patients: 275 bacterial and 31 fungal. Most patients (119; 85%) had dual-organism coinfections, the most common of which was the combination of *Staphylococcus epidermidis* with *Kocuria* spp., while 17 patients (12%) had triple-organism infections, the most common of which was *Staphylococcus epidermidis* with *Kocuria rosea* and *Granulicatella elegans* or with filamentous fungus, 3 patients (2%) had four (*Serratia marcescens* with *Staphylococcus epidermidis*, *Aeromonas caviae,* and *Kocuria rosea*; *Aspergillus* sp. with *Kocuria rosea*, *Staphylococcus epidermidis,* and *Staphylococcus aureus,* and *Staphylococcus epidermidis* with *Fusarium* sp., *Staphylococcus warneri,* and *Staphylococcus capitis*), and 1 patient (1%) had five (*Staphylococcus epidermidis* with *Staphylococcus* spp., *Staphylococcus capitis, Staphylococcus hominis,* and *Fusarium* sp.).

**Figure 2:**
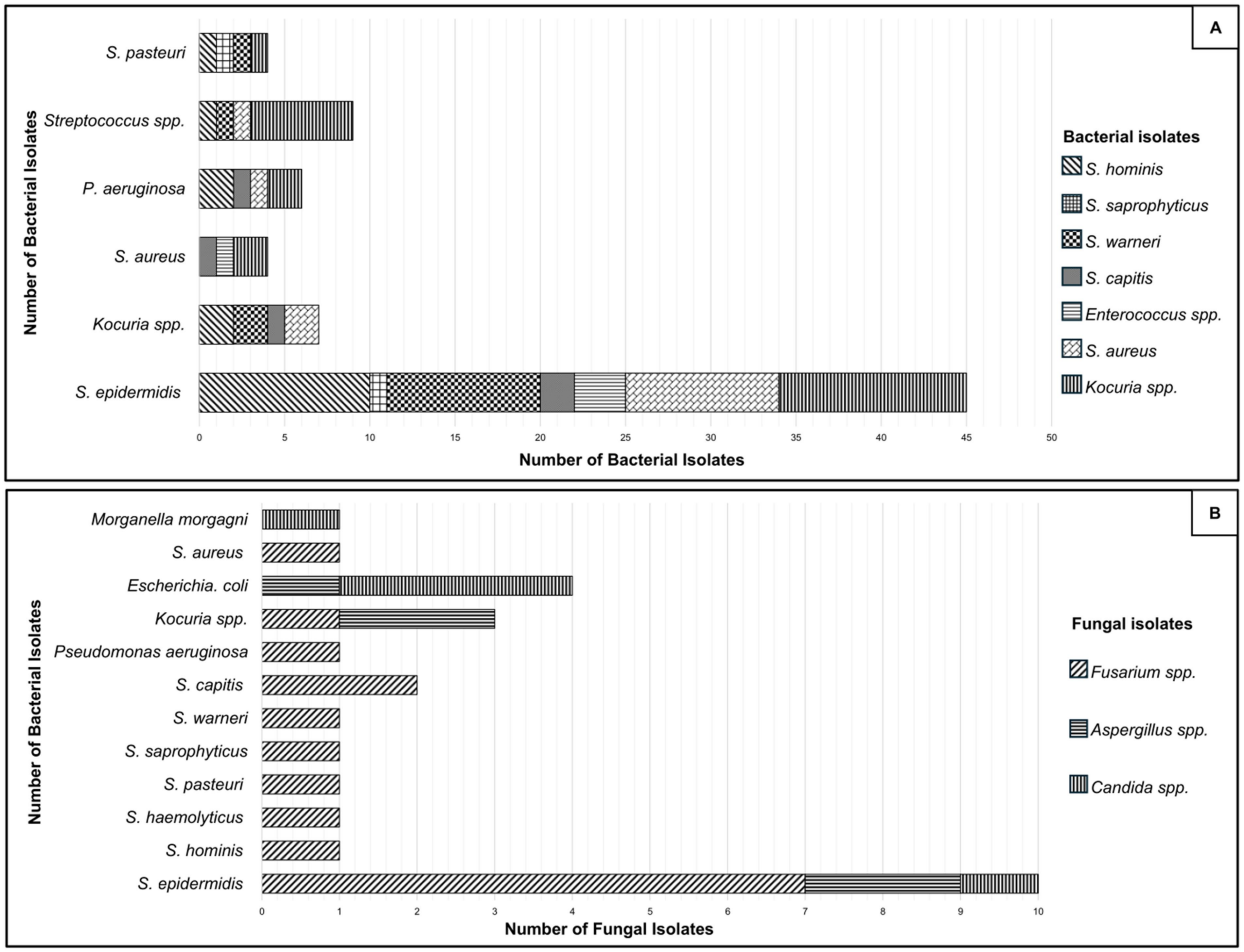
Distribution of bacterial isolates from corneal cultures in cases of bacterial-bacterial **(A)** and bacterial-fungal **(B)** coinfections. In which they are considered as coagulase negative Staphylococci (CNS): *Staphylococcus hominis*, *Staphylococcus warneri*, *Staphylococcus auricularis*, *Staphylococcus capitis*, *Staphylococcus caprae*, *Staphylococcus saprophyticus*, *Staphylococcus cohnii*, *Staphylococcus chromogenes*, *Staphylococcus intermedius*, *Staphylococcus lentus*, *Staphylococcus lugdunensis*, *Staphylococcus pasteuri*, *Staphylococcus simulans* and *Staphylococcus haemolyticus*, were the most frequent group. Fungal isolates were predominantly *Fusarium* spp.

In the case of bacteria, Gram-positive bacteria were predominant (85% of all bacterial cultures) (Table 2). The most common bacterial species was *Staphylococcus epidermidis* (28%), followed by other coagulase-negative *staphylococci* (CNS; 18%). Among fungal isolates, filamentous fungi predominated (61%), with *Fusarium* spp. being the most frequently identified (52%), followed by *Candida* spp. (16%) (Table 3). There were 109 (78%) cases of mixed bacterial with bacterial keratitis infection. The most frequent were *S. epidermidis* + other CNS (21%), followed by *S. epidermidis* + *Kocuria* spp. (10%), and *S. epidermidis* + *Staphylococcus aureus* (10%) (Figure 2A). Among bacterial-fungal coinfections 31 (22%), *S. epidermidis* + *Fusarium* (23%) and CNS + *Fusarium* (23%) were most common, followed by *Escherichia coli* + *Candida* spp. (10%) (Figure 2B).

**Table 2:** Bacterial isolates from corneal cultures of eyes diagnosed with mixed bacterial and fungal keratitis.

Name of the bacterial isolates	No. isolates (%) (*n* = 275)
Gram-positive bacteria	**234 (85%)**
*Staphylococcus* species	147 (53%)
*S. epidermidis*	78 (28%)
*S. aureus*	18 (7%)
*Other CNS*	50 (18%)
*S. hominis*	19 (7%)
*S. warneri*	13 (5%)
*S. capiti*	6 (2%)
*S. pasteuri*	5 (2%)
*S. saprophyticus*	2 (1%)
*S. lugdunensis*	2 (1%)
*S. lentus*	1 (0.4%)
*S. chromogenes*	1 (0.4%)
*S. cohnii*	1 (0.4%)
Other	1 (0.4%)
*Kocuria* species	30 (11%)
*K. rosea*	21 (8%)
*K. kristinae*	6 (2%)
*K. varians*	3 (1%)
*Streptococcus* species	20 (7%)
*S. pneumoniae*	6 (2%)
*S. parasanguini*	1 (0.4%)
*S. oralis*	1 (0.4%)
*S. mitis*	2 (1%)
Other	10 (4%)
*Enterococcus* species	8 (3%)
*E. faecalis*	6 (2%)
*E. faecium*	2 (1%)
Other gram-positive	19 (7%)
Gram-negative bacteria	**41 (15%)**
*Pseudomonas* species	11 (4%)
*P. aeruginosa*	10 (4%)
*P. stutzeri*	1 (0.4%)
*Serratia* species	6 (2%)
*S. marcescens*	6 (2%)
*Klebsiella* species	3 (1%)
*K. oxytoca*	2 (1%)
*K. pneumoniae*	1 (0.4%)
*Acinetobacter* species	3(1%)
*A. Iwoffi*	2 (1%)
*A. haemolyticus*	1 (0.4%)
Other gram-negative	18 (7%)

Other CNS: other coagulase-negative staphylococci. Bold text indicates group/category labels.

**Table 3:** Fungal isolates from corneal cultures of eyes diagnosed with mixed bacterial and fungal keratitis.

Name of the fungal isolates	No. isolates (%) (*n* = 31)
Filamentous fungus	**26 (61%)**
*Fusarium*	16 (52%)
*Aspergillus*	4 (10%)
*Paecillomyces*	2 (7%)
*Scedosporium*	1 (3%)
*Alternaria*	1 (3%)
*Curvularia*	1 (3%)
Unidentified filamentous fungus	2 (7%)
*Candida* spp.	5 (16%)

Bold text indicates group/category labels.

Seasonal trends were observed in the distribution of coinfection type. Among bacterial coinfections, spring accounted for 30 cases (27%), summer for 25 cases (23%), autumn for 28 cases (26%), and winter for 26 cases (24%). In contrast, bacterial-fungal coinfections were more frequent in spring, with 12 cases (39%), followed by winter with 8 cases (26%), summer with 6 cases (19%), and autumn with 5 cases (16%). A Chi-square test was performed to compare the seasonal distribution of bacterial-bacterial vs. fungal-bacterial coinfections. The test showed no significant difference (*χ*^2^ = 2.120, *p* = 0.547), indicating that the seasonal distribution did not differ significantly between both coinfection types. Institutional data from 1,770 monomicrobial keratitis cultures collected at the same institution between 2012 and 2020, 1,619 bacterial and 129 fungal were reviewed for contextual reference. This study showed a predominance of Gram-positive bacteria (1,399 of 1,619; 86%), followed by Gram-negative isolates (220 of 1,619; 13.6%) and fungi 129 (7%). The most frequent bacterial species were *Staphylococcus epidermidis* 729 (45%), other CNS 229 (14%), *Kocuria* spp. 125 (8%), *Pseudomonas aeruginosa* 118 (7%), and *Staphylococcus aureus* 112 (7%). Among fungi, *Fusarium* spp. (51%), *Aspergillus* spp. (20%), and *Candida* spp. (19%) were the main species identified.

### 3.3 Antibiogram

**Figure 3:**
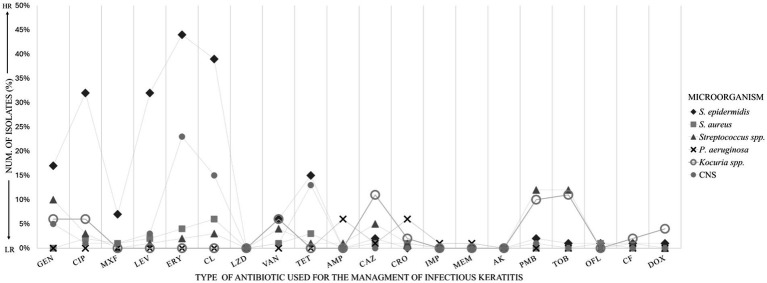
Sensitive antibiotic profiles of bacterial isolates. Linear bars show bacterial sensitivity to antibiotics used for infectious keratitis. Complete sensitivity is shown to the antibiotics LZD and AK. High resistance to ERY, CL, LEV and CIP by *S. epidermidis* is shown. HR, high resistance; LR, low resistance; GEN, gentamicin; CIP, ciprofloxacin; MXF, moxifloxacin; LEV, levofloxacin; ERY, erythromycin; CL, clindamycin; LZD, linezolid; VAN, vancomycin; TET, tetracycline; AMP, ampicillin; CAZ, ceftazidime; CRO, ceftriaxone; IMP, imipenem; MEM, meropenem; AK, amikacin; PMB, polymyxin; TOB, tobramycin; OFL, ofloxacin; CF, cephalothin; DOX, doxycycline; other CNS: other coagulase-negative staphylococci.

Antibiotic susceptibility testing (Figure 3) was performed. Nine of 14 (64%) isolates were cefazolin resistant; 28 of 50 (56%) isolates were polymyxin resistant; 81 of 156 (52%) isolates were erythromycin resistant; 70 of 153 (46%) isolates were clindamycin resistant; 29 of 65 (45%) isolates were ceftazidime resistant; 39 of 88 (44%) isolates were tobramycin resistant; 20 of 70 (29%) isolates were ampicillin resistant; 40 of 154 (26%) isolates were tetracycline resistant; 40 of 154 (26%) isolates were levofloxacin resistant; 53 of 242 (22%) isolates were ciprofloxacin resistant; 47 of 239 (20%) isolates were gentamicin resistant; 16 of 91 (18%) isolates were ceftriaxone resistance; 2 of 12 (16%) isolates were ofloxacin resistance; 28 of 218 (13%) isolates were vancomycin resistance; 2 of 16 (13%) isolates were cephalothin resistance; 15 of 174 (9%) isolates were moxifloxacin resistance; 1 of 15 (7%) isolates were doxycycline resistance; 3 of 75 (4%) isolates were imipenem resistance; 1 of 26 (4%) isolates were meropenem resistance. All isolates were sensitive to linezolid and amikacin.

In addition to general resistance rates, seasonal variation in antimicrobial resistance was also noted. Cefazolin-resistant strains were most frequently identified during winter (36%). In spring, several resistances predominated, including polymyxin (20%), erythromycin (17%), clindamycin (17%), ceftazidime (17%), tobramycin (16%), levofloxacin (8%), ciprofloxacin (7%), gentamicin (7%), ofloxacin (7%), and moxifloxacin (5%).

Resistance to ampicillin peaked in summer (11%), along with doxycycline (6%) and ceftriaxone (7%). Tetracycline resistance was more prevalent in autumn (8%), as was cephalothin resistance (15%). Finally, imipenem and meropenem resistance appeared predominantly in winter, each accounting for (4%) of isolates.

### 3.4 Gram-negative organism susceptibility

Gram-negative bacteria exhibited high susceptibility to amikacin and ofloxacin (100%), gentamicin (33 of 34; 97%), imipenem (28 of 29; 97%), and meropenem (23 of 24; 96%). However, resistance to ampicillin (13 of 23; 57%), ceftriaxone (8 of 31; 26%), polymyxin (1 of 4; 25%) and tobramycin (7 of 28; 25%) were observed, particularly among *Pseudomonas aeruginosa*, which was 100% susceptible to amikacin, gentamicin, ciprofloxacin, moxifloxacin, and tobramycin. Moreover, *P. aeruginosa* (86%) were resistant to ampicillin 6 of 7 and ceftriaxone 6 of 7, followed by ceftazidime (2 of 7; 29%) were resistant and imipenem (1 of 8; 13%) were resistant.

In the institutional monomicrobial dataset, *P. aeruginosa* demonstrated high susceptibility to imipenem and moxifloxacin (113/118; 96%), but resistance to ceftriaxone and ampicillin in (98/118; 83%). No statistically significant difference was observed in ceftriaxone, ampicillin and imipenem resistance between *P. aeruginosa* isolates from coinfections and monomicrobial keratitis cases (*p* = 1.00 and *p* = 0.33 respectively; Fisher’s exact test). However, resistance to ceftazidime was identified more frequently among coinfection isolates (*p* = 0.02; Fisher’s exact test).

### 3.5 Gram-positive organism susceptibility

Linezolid showed 100% of susceptibility to Gram-positive isolates, followed by (33 of 34; 97%) who were susceptible to ampicillin, (85 of 94; 90%) whose isolates were susceptible to moxifloxacin, (7 of 8; 89%) were susceptible to doxycycline, and (124 of 146; 85%) who were susceptible to vancomycin. Thirty two of 51 (63%) were resistant to tobramycin, (48 of 96; 50%) were resistant to erythromycin, and (48 of 97; 49%) were resistant to clindamycin. *S. epidermidis* was (71 of 77; 92%) susceptible to vancomycin and (67 of 74; 91%) to moxifloxacin. Other CNS isolates exhibited high susceptibility to ciprofloxacin (98%), moxifloxacin (98%), and vancomycin (98%). However, *S. epidermidis* was resistant to ceftazidime (1 of 3; 67%), erythromycin (33 of 77; 57%) and clindamycin (38 of 77; 51%). Other CNS was resistant to erythromycin (25 of 48; 48%), clindamycin (33 of 48; 31%) and tetracycline (35 of 48; 27%).

In the institutional monomicrobial dataset, *S. epidermidis* were characterized by (678/729; 93%) to vancomycin and linezolid (100%), with resistance to ciprofloxacin (385/729; 53%), erythromycin (381/729; 52%) and clindamycin (384/729; 48%). Other CNS showed (211/229; 92%) susceptibility to vancomycin and resistance to erythromycin (120/229; 52%), clindamycin (77/229; 34%), and tetracycline (61/229; 27%). No statistically significant difference was observed in erythromycin and clindamycin resistance between *S. epidermidis* isolates from coinfections and monomicrobial keratitis cases (*p* = 0.12 and *p* = 0.81 respectively; Fisher’s exact test). No statistically significant difference was observed in erythromycin resistance between other CNS isolates from coinfections and monomicrobial keratitis cases (*p* = 1.00; Fisher’s exact test). Resistance to clindamycin and tetracycline appeared more frequently among CNS isolates from coinfection cases (both *p* = <0.0001, Fisher’s exact test).

### 3.6 Clinical features of the most frequent coinfections patterns

Among the most frequent coinfection patterns (e.g., *S. epidermidis*- other CNS, *S. epidermidis*-*Kocuria*, *S. epidermidis-Fusarium* and CNS-*Fusarium*), further clinical analysis was conducted. Average final visual acuity values were calculated only from patients with complete follow-up data.

In the *S. epidermidis*- other CNS group, 13% of patients had a history of diabetes, 13% had previous ocular surgery, 13% used corticosteroids, and 22% required surgical intervention due to treatment failure. The average final visual acuity was 8.9 logMAR (*n* = 6).

In the *S. epidermidis-Kocuria* group, 18% of patients had a history of diabetes; 45% had previous ocular surgery, 18% used corticosteroids, and 18% required surgical intervention due to treatment failure. The average final visual acuity was 9.2 logMAR of (*n* = 5).

In the *S. epidermidis-Fusarium* group, 14% of patients had a history of diabetes, none had previous ocular surgery, none received corticosteroid therapy, and 29% required surgical intervention due to treatment failure. The average final visual acuity was 1.5 logMAR of (*n* = 4).

In the CNS-*Fusarium* group, 14% of patients had a history of diabetes, 14% had previous ocular surgery, none received corticosteroid therapy, and 29% required surgical intervention due to treatment failure. The average final visual acuity was 3.2 logMAR of (*n* = 2).

Regarding seasonal distribution, bacterial–bacterial coinfections such as *Staphylococcus epidermidis*–other CNS were most frequent in spring (39%), and *S. epidermidis–Kocuria* coinfections also peaked during spring (45%). Among bacterial–fungal coinfections, *S. epidermidis–Fusarium* showed a balanced distribution across spring, summer, and winter (29% each), while CNS–*Fusarium* coinfections were most prevalent in spring, summer, and autumn (29% each).

### 3.7 Treatment outcomes

Initial treatment was determined empirically. It consisted of antibiotic and antifungal therapies guided by medical history and clinical findings, and was later adjusted based on clinical response, culture results, and antibiotic susceptibility (when available).

Topical moxifloxacin 0.5% was administered to 63% of patients every 1–2 h during the acute phase. Oral doxycycline (100 mg twice daily) was prescribed to control inflammation and was given to 31% of patients. Antifungal therapy included natamycin 5% eye drops, administered every 1–2 h in 15% of patients, and oral itraconazole (200 mg daily), used in 9% of patients with deep stromal involvement. Corticosteroids (e.g., prednisolone acetate 1%) were introduced in 20% of patients, but only after epithelial healing and under close clinical supervision. Treatment duration ranged from 2 to 6 weeks, depending on the severity of the infection and the clinical response. Treatment failure occurred in 56 patients (40%), of whom 27 required surgical intervention (including penetrating keratoplasty and amniotic membrane transplantation). Three patients declined surgery. Seventh patients with fungal microorganisms were treated with corticosteroids of which 6 (19%) had treatment failure. Surgical failure occurred in 6 patients (4%), while 84 patients (60%) responded successfully to initial pharmacological therapy. Corneal re-epithelialization occurred in only 41% of patients, with a mean duration of 48.4 days (SD ± 28.8) during the follow-up period. A statistically significant difference was observed between treatment success and failure groups (*p* < 0.001; chi-squared test).

Patients underwent various surgeries, including penetrating keratoplasty (16 patients; 12%), amniotic membrane transplantation (9 patients; 6%), and evisceration (5 patients; 4%). Visual acuity in presentation ranged from 20/20 to light perception. When initially evaluated, 67 (48%) patients could see hand movement or worse, 16 (11%) patients could see finger count, 23 (16%) patients had 20/800 to 20/200 vision, and 34 (24%) patients had 20/125 vision or better. At the time of the last follow-up, the visual acuity of 34 patients (24%) was hand movement or worse, 5 (4%) patients could see a finger count, 11 (8%) patients had 20/800 to 20/200 vision, and 14 (10%) patients had 20/125 vision or better; 76 patients did not return for follow-up. There was a difference between pre-treatment visual acuity (1.48 ± 1.00) and the post-treatment visual acuity (1.58 ± 1.04; *p* = 0.27, paired t-test). The ocular, systemic, and environmental conditions at the time of the corneal swab were also defined in cases mixed bacterial-bacterial and bacterial-fungal keratitis; the results are provided in (Table 1).

## 4 Discussion and conclusion

This retrospective and descriptive study characterizes microbial coinfections in infectious keratitis and provides a contextual comparison with institutional monomicrobial isolates collected during the same period.

Microbial coinfections can be classified into two types: negative, where one microorganism inhibits the growth of the other, and positive, where a synergistic interaction enhances mutual survival. For instance, *Candida albicans* has been shown to protect *Staphylococcus aureus* through its biofilm components, and vice versa (Ramírez Granillo et al., 2015). Similarly, in herpetic corneal ulcers, increased microbial adherence – by both bacteria and fungi – has been reported, leading to greater corneal surface damage and antimicrobial resistance (Tuft, 2006). Moreover, in murine models, structural damage to the cornea has been observed following coinfections with *Staphylococcus aureus* and *Fusarium falciform* (Ponce-Angulo et al., 2020).

Mixed microbial keratitis presents a diagnostic and therapeutic challenge due to the complex interactions between pathogens, overlapping clinical features, and frequent delays in microbiological confirmation. In our 8-year retrospective study, we identified 140 cases of confirmed coinfection, comprising both bacterial–bacterial and bacterial–fungal keratitis. The increasing frequency of such cases, also reported in recent literature (Boonpasart et al., 2002; Leck et al., 2002), highlights the need for heightened clinical awareness, particularly in tertiary referral centers were patients often present late or after initial treatment failure.

Our findings showed a predominance of *Staphylococcus epidermidis* and coagulase-negative staphylococci (CNS) among bacterial isolates, and *Fusarium* spp. among fungi. *S. epidermidis*, while often dismissed as a commensal, has been implicated as a true pathogen in numerous keratitis cases, particularly those associated with prior surgery or device use (Pate et al., 2006; Tam et al., 2017; Haro-Morlett et al., 2024). The presence of these organisms in coinfection scenarios supports the hypothesis that biofilm formation and epithelial compromise play key roles in disease pathogenesis (Thomas, 2003; Ansari et al., 2013).

Risk factors in our cohort mirrored those reported in other regions: contact lens use in younger patients and prior ocular surgery or diabetes in older individuals (Boonpasart et al., 2002; Bourcier et al., 2017; Green et al., 2008). Notably, 34% of our cases had a history of surgery, aligning with reports that corneal incisions and altered tear dynamics facilitate microbial colonization (Leck et al., 2002). Diabetes mellitus was present in 20%, which may reflect impaired epithelial healing and immune dysregulation (Khoo et al., 2020).

A key observation was that fungal coinfections, though fewer in number (n = 31), were associated with more severe presentations and higher failure rates. Patients in this subgroup had significantly worse visual acuity at presentation and were more likely to require surgical intervention. These findings are consistent with those of Bourcier et al. (2017) and Donovan et al. (2022), who emphasized the delayed diagnosis and slow therapeutic response in fungal keratitis. Moreover, the injudicious use of corticosteroids—reported in 20% of our patients—may worsen outcomes in fungal cases, as noted by Sharma et al. (2022).

Seasonal variation in the distribution of pathogens was also observed. Studies from Japan and the United Kingdom have shown that *Pseudomonas aeruginosa* and gram-negative organisms tend to occur during warmer months, whereas gram-positive bacteria and filamentous fungi are more frequently isolated in cooler or transitional seasons (Cao et al., 2023; Yamada et al., 2025; Erdem et al., 2024; Walkden et al., 2018). In our study, *S. epidermidis*– other CNS coinfections were recorded mainly during spring, while bacterial–fungal combinations such as *S. epidermidis*–*Fusarium* were identified throughout the year. These observations suggest potential environmental or exposure-related influences on pathogen dynamics.

Antimicrobial resistance patterns identified in this cohort are consistent with national and international reports (Hernandez-Camarena et al., 2015; Ting et al., 2021). Among *S. epidermidis* isolates, resistance to erythromycin (57%), clindamycin (51%), and fluoroquinolones (42–43%) was commonly observed. *Pseudomonas aeruginosa* isolates showed susceptibility to amikacin, tobramycin, and fluoroquinolones but frequent resistance to *β*-lactams is a trend also reported elsewhere (Donovan et al., 2022; Ting et al., 2021). These findings underscore the importance of continuous local surveillance to guide empirical therapy and support antimicrobial stewardship in ophthalmology.

From a clinical outcome perspective, our data revealed that 60% of patients responded to medical therapy alone, whereas 40% required surgical management, including PKP and amniotic membrane transplantation. The rate of surgical intervention, while high, is comparable to other tertiary center reports and may reflect the delayed presentation seen in coinfection cases (Leck et al., 2002; Donovan et al., 2022).

To provide additional context, we compared our findings with data from 1,770 monomicrobial keratitis isolates collected at the same institution between 2012 and 2020 were reviewed (Barragán et al., 2022). These monomicrobial data were included solely as background context and not for inferential comparison- Similar microbial profiles were observed, with *S. epidermidis* as the predominant bacterium (45%), followed by other CNS, *Kocuria*, and *Pseudomonas aeruginosa*, along with comparable resistance trends. This contextual inclusion highlights the boarder epidemiological environment in which coinfections occur, without implying statistical association or causality. While resistance to ceftazidime and certain antibiotics appear more frequently among coinfection isolates, these findings should be interpreted descriptively. They suggest potential microbial interaction that may influence resistance expression; however, confirmatory studies are required to establish causality.

This study has several limitations. Its retrospective design may introduce selection or reporting bias, and some microorganisms may have been undetected due to prior antimicrobial use or limitations of conventional culture. The absence of antifungal susceptibility testing and molecular diagnostics, such as PCR or MALDI-TOF, restricted the detection of fastidious organisms. Nevertheless, culture-based methods remain the standard of care in many resource-limited environments, and our results reflect real-world clinical practice in such settings.

Despite these limitations, this study represents one of the most detailed institutional descriptions of microbial coinfection keratitis in Latin America. By characterizing clinical features, microbiological patterns, and antimicrobial susceptibility profiles, it contributes to understanding the spectrum of these complex infections. The findings underline the importance of early microbiological investigation and tailored therapy guided by local resistance data.

Future prospective or case–control studies directly comparing coinfection and monomicrobial cases are warranted to clarify differences in clinical outcomes, antimicrobial response, and possible synergistic interactions among pathogens.

### 4.1 Conclusion

This retrospective study characterizes microbial coinfections in infectious keratitis as a clinically significant and increasingly recognized entity. Coinfections were characterized by the frequent involvement of *S. epidermidis*, CNS, and *Fusarium* spp., together with notable antimicrobial resistance. Despite sharing a similar microbial spectrum with monomicrobial keratitis, coinfections appear to present greater clinical complexity and higher rates of therapeutic failure.

These findings emphasize the importance of early microbiological investigation, individualized therapy guided by local resistance patterns, and cautious use of corticosteroids in uncertain etiologies. Future multicenter or case–control studies integrating molecular methods are warranted to clarify the clinical impact and synergistic mechanisms of polymicrobial keratitis.

## Data Availability

The raw data supporting the conclusions of this article will be made available by the authors, without undue reservation.
